# Toward Understanding Death

**DOI:** 10.1093/cid/ciab809

**Published:** 2021-12-15

**Authors:** Samantha B Dolan, Barbara E Mahon, Scott F Dowell, Anita Zaidi

**Affiliations:** Bill & Melinda Gates Foundation, Seattle, Washington, USA

**Keywords:** mortality, pathology, cause-of-death, population health

## Abstract

Evidence-based approaches to preventing child death require evidence; without data on common causes of child mortality, taking effective action to prevent these deaths is difficult at best. Minimally invasive tissue sampling (MITS) is a potentially powerful, but nascent, technique to obtain gold standard information on causes of death. The Gates Foundation committed to further establishing the methodology and obtain the highest quality information on the major causes of death for children under 5 years. In 2018, the MITS Surveillance Alliance was launched to implement, refine, and enhance the use of MITS across high mortality settings. The Alliance and its members have contributed to some remarkable opportunities to improve mortality surveillance, and we have only just begun to understand the possibilities on larger scales. This supplement showcases studies conducted by MITS Surveillance Alliance members and represents a significant contribution to the cause-of-death literature from high mortality settings.

Losing a child is devastating; even worse is losing an infant or child and not knowing why. This tragedy is compounded in poor countries where stillbirth, neonatal, infant, and child mortality is common, but reliable diagnosis of causes of death are rare. Evidence-based approaches to preventing pediatric death require objective data; without data on common causes of child mortality, taking effective action to prevent these deaths is difficult at best.

In 2015, the Bill and Melinda Gates Foundation committed to generating more definitive evidence on the causes of child mortality, investing in minimally invasive tissue sampling (MITS), a potentially powerful, but nascent, technique to obtain high-quality information on the causes of death. Conventional medical autopsies remain the gold standard for cause-of-death attribution; however, a growing number of studies provide evidence that MITS is a comparable technique [1–4]. In MITS, biopsy needles are used for post-mortem sampling of key organs for pathology, microbiology, and other testing to establish the cause of death without a full autopsy. MITS, also called minimally invasive autopsy, was largely developed by the ISGlobal research group to provide a robust but simplified alternative to conventional autopsies to make cause-of-death attribution more accessible in low-income countries [1, 5, 6]. The foundation committed to further establishing the MITS methodology and obtain the highest quality information on the major causes of death for children under 5 years. To be successful, MITS needed not only to yield definitive data but also to be acceptable to bereaved families and communities. The foundation launched a 20-year investment in the Child Health and Mortality Prevention Surveillance (CHAMPS) network, spanning high mortality areas in Africa and South Asia, and using MITS for pathology-based cause-of-death determination. CHAMPS has yielded insights never before available about the causes of deaths in neonates, infants, and young children in these sites [7, 8].

As the value of definitive pathology-based cause of death information in high mortality areas became more apparent, other research groups approached the foundation for technical advice and support for MITS. Therefore, in 2018 the foundation funded the MITS Surveillance Alliance. Built on the experience of ISGlobal, CHAMPS, and the Project to Understand and Research Preterm Pregnancy Outcomes and Stillbirths in South Asia, and facilitated by Research Triangle Institute (RTI) International, the Alliance’s aim was to implement, refine, and enhance the use of MITS across high mortality settings [9]. As the articles in this supplement illustrate, the Alliance has supported the expansion of MITS for mortality surveillance and research, facilitated the development and promulgation of best practices, and developed a dynamic community of practice. Since its inception, the Alliance has made it possible for practitioners to establish MITS in new settings. It has trained pathologists, assembled and distributed MITS equipment kits, and provided forums for exchange of learnings. The Alliance has developed a training hub at Kenyatta National Hospital in Nairobi, localizing in-country capacity. The Alliance’s subcommittees have contributed to important advances in cause-of-death attribution. For example, the undernutrition subcommittee developed new guidance for evaluating undernutrition in the causal chain of events leading to childhood death, and the social and behavioral sciences subcommittee has guided the family consent process across cultures. The Alliance played an unexpected role early in the coronavirus disease 2019 (COVID-19) pandemic, developing guidelines for MITS in COVID-19 cases and establishing their feasibility, adequacy, and safety [10].

The Alliance and its members have contributed to some remarkable opportunities to improve mortality surveillance. Improvements in tissue sampling practices and application of machine learning techniques to help automate data interpretation may offer substantial efficiencies and can help to solidify the technique as a game-changer for mortality surveillance, within both low-and high-income settings. User-friendly guidance and documentation can increase MITS accessibility and consistency in new sites, and research on acceptability can promote uptake in facility and community-based settings. Improvements in Bayesian methods for predicting underlying causes of death raise the possibility of using MITS-informed causes of death to calibrate verbal autopsy data to improve cause of death attribution and estimation in populations where MITS is not performed [11]. Given the expanding interest, with sites specially requesting additional resources to support long-term use of the methodology to inform local mortality surveillance activities, alternative and diverse funding models may be considered. Continuing to explore how MITS cases can be best leveraged will be an ongoing theme in the coming years. We have only just begun to understand the possibilities on larger scales.

This supplement showcases studies conducted by MITS Surveillance Alliance members and represents a significant contribution to the cause-of-death literature from high mortality settings. Each MITS case brings us closer to understanding the major causes of mortality. By continuing to invest in techniques that can, with high acceptability, identify causes of death in low-resource settings, and develop solutions to address them we move closer to a world where children, families, and communities no longer needlessly suffer from preventable deaths.

## Notes

***Financial sources***. This work was supported by the Bill & Melinda Gates Foundation (grant number OPP1180554).

***Supplement sponsorship.*** This supplement is sponsored by MITS Surveillance Alliance Secretariat, led by RTI International, with funding from the Bill and Melinda Gates Foundation.

***Potential conflicts of interest***. S. B. D., S. F. D., and A. Z. are employed by the funder of the MITS (Minimally Invasive Tissue Sampling) Surveillance Alliance. B. E. M. was previously employed by the funder. All other authors report no potential conflicts. All authors have submitted the ICMJE Form for Disclosure of Potential Conflicts of Interest. Conflicts that the editors consider relevant to the content of the manuscript have been disclosed.

## References

[1] Castillo P, Martínez MJ, Ussene E, et al. Validity of a minimally invasive autopsy for cause of death determination in adults in Mozambique: an observational study. PLoS Med 2016; 13:e1002171. 27875530 10.1371/journal.pmed.1002171 PMC5119723

[2] Bassat Q, Castillo P, Martínez MJ, et al. Validity of a minimally invasive autopsy tool for cause of death determination in pediatric deaths in Mozambique: an observational study. PLoS Med 2017; 14:e1002317. 28632739 10.1371/journal.pmed.1002317 PMC5478091

[3] Menendez C, Castillo P, Martínez MJ, et al. Validity of a minimally invasive autopsy for cause of death determination in stillborn babies and neonates in Mozambique: an observational study. PLoS Med 2017; 14:e1002318. 28632735 10.1371/journal.pmed.1002318 PMC5478138

[4] Castillo P, Hurtado JC, Martínez MJ, et al. Validity of a minimally invasive autopsy for cause of death determination in maternal deaths in Mozambique: an observational study. PLoS Med 2017; 14:e1002431. 29117196 10.1371/journal.pmed.1002431 PMC5695595

[5] Castillo P, Ussene E, Ismail MR, et al. Pathological methods applied to the investigation of causes of death in developing countries: minimally invasive autopsy approach. PLoS One 2015; 10:e0132057. 26126191 10.1371/journal.pone.0132057 PMC4488344

[6] Bassat Q, Castillo P, Alonso PL, Ordi J, Menéndez C. Resuscitating the dying autopsy. PLoS Med 2016; 13:e1001927. 26756992 10.1371/journal.pmed.1001927 PMC4710495

[7] Salzberg NT, Sivalogan K, Bassat Q, et al; Child Health and Mortality Prevention Surveillance (CHAMPS) Methods Consortium. Mortality surveillance methods to identify and characterize deaths in child health and mortality prevention surveillance network sites. Clin Infect Dis 2019; 69:262–73. 10.1093/cid/ciz599 PMC6785672 31598664

[8] Nkengasong J, Gudo E, Macicame I, et al. Improving birth and death data for African decision making. Lancet Glob Health 2020; 8:e35–6. 31839138 10.1016/S2214-109X(19)30397-3

[9] McClure EM, Saleem S, Goudar SS, et al. The project to understand and research preterm pregnancy outcomes and stillbirths in South Asia (PURPOSe): a protocol of a prospective, cohort study of causes of mortality among preterm births and stillbirths. Reprod Health 2018; 15:89. 29945651 10.1186/s12978-018-0528-1 PMC6020001

[10] Rakislova N, Marimon L, Ismail MR, et al. Minimally invasive autopsy practice in COVID-19 cases: biosafety and findings. Pathogens 2021; 10:412. 33915771 10.3390/pathogens10040412 PMC8065952

[11] Datta A, Fiksel J, Amouzou A, Zeger SL. Regularized Bayesian transfer learning for population-level etiological distributions. B-iostatistics 2020:kxaa001. 10.1093/biostatistics/kxaa001 PMC8511959 32040180

